# Supplementary material for: miR-129a-3p Inhibits PEDV Replication by Targeting the EDA-Mediated NF-κB Pathway in IPEC-J2 Cells
Source: Int J Mol Sci. 2021 Jul 29;22(15):8133. doi: 10.3390/ijms22158133 (PMC8347983; doi:10.3390/ijms22158133)
Supplement: Supplementary file 1 [file ijms-22-08133-s001.zip › supplementary table1.pdf]

| 基因 | 引物序列（5’→ 3’） | GenBank 登陆号 | 产物长度，bp |
|---|---|---|---|
| *EDA* | F:CAAGGGTCAGCCATCC<br>R: GAAGTAGGTGCCGTCCA | XM_005657816.3 | 141 |
| *PEDV-M* | F: AGGTCTGCATTCCAGTGCTT<br>R: GGACATAGAAAGCCCAACCA | AF353511.1 | 216 |
| *ssc-miR-129a-3p* | F:AAGCCCTTACCCCAAAAAGCAT | NR_038576.1 | |
| *U6* | F:ATAGATCTAGGAGGACTCCAGGGAC<br>R: CTGAATTCGGGTCTTCTCAGAGG | NR_138085.1 | |
| *GAPDH* | F:ACATCATCCCTGCTTCTACTGG<br>R:CTCGGACGCCTGCTTCAC | AF017079.1 | 188 |
| *CARD11* | F:GCGCCCGAGATGGATGACTAC<br>R:ATGGCAGCATGGGAGCGTTAA | XM_021086018.1 | 190 |
| *OASL* | F:TGGAAGGGCCCATCACTTTC<br>R:CTGGCTTTCACGTACTTCAGG | NM_001031790.1 | 229 |
| *PTGS2* | F:AGCAGGCTGATACTGATAGGAGA<br>R:AGCAGCTCTGGGTCAAACTT | NM_214321.1 | 229 |
| *CXCL8* | F:TGGACCCCAAGGAAAAGTGG<br>R:TGTTGTTGCTTCTCAGTTCTCTTC | NM_213867.1 | 70 |
| *IL16* | F:ATGCGAAGACCAGTGACGAGG<br>R:GGCCAGCATGAGGTCAGGAGT | NM_213751.2 | 161 |

| | | | |
|---|---|---|---|
| *IL19* | F:TGGGTACGATGCTCTTCCTG<br>R:ATGGTGTCCTTAGCTTGGATG | XM_003130464.3 | 130 |

**Supplementary table S1**
