# Supplementary figures and images for: miR-129a-3p Inhibits PEDV Replication by Targeting the EDA-Mediated NF-κB Pathway in IPEC-J2 Cells
Source: Int J Mol Sci. 2021 Jul 29;22(15):8133. doi: 10.3390/ijms22158133 (PMC8347983; doi:10.3390/ijms22158133)

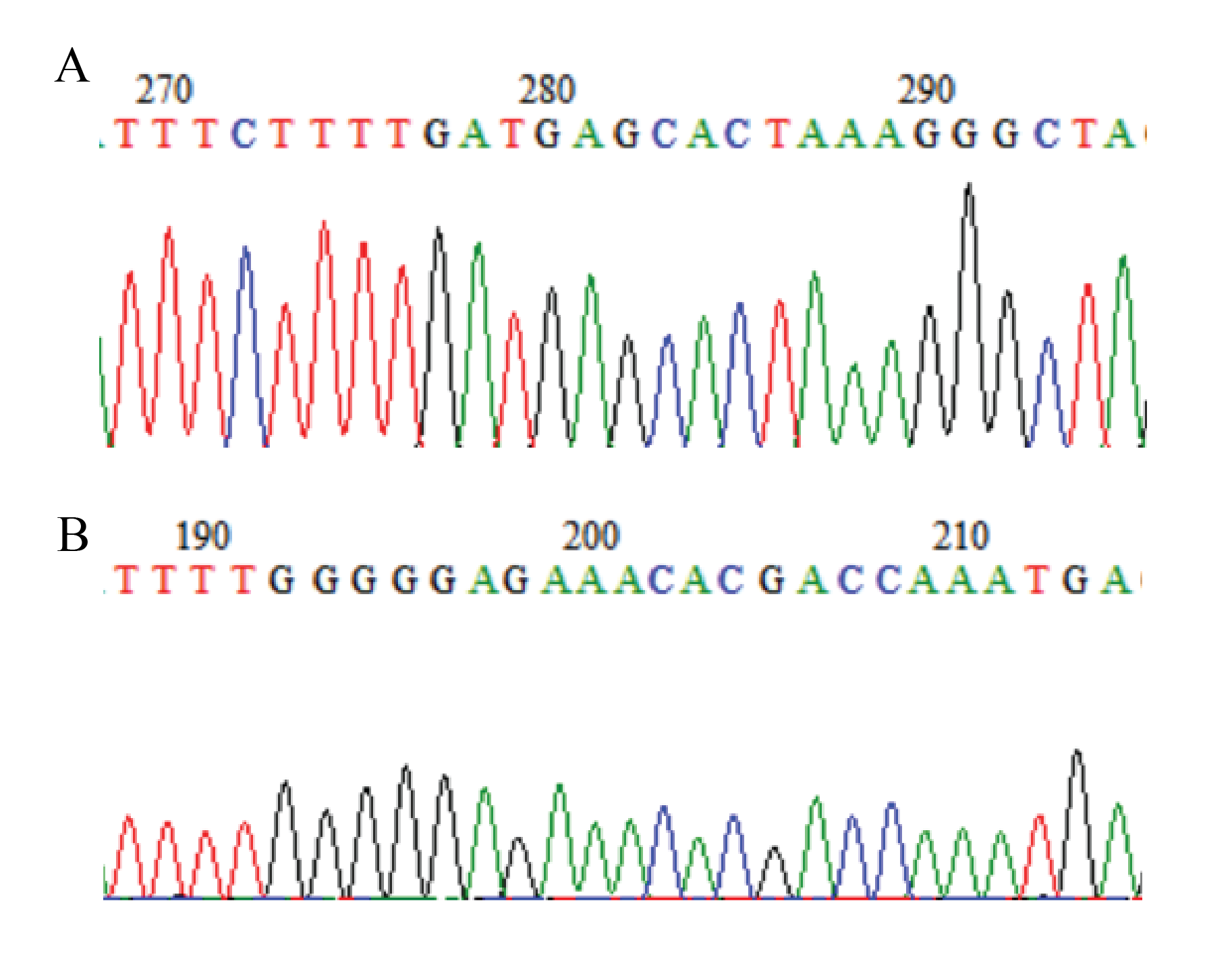

Supplement: Supplementary file 1 [file ijms-22-08133-s001.zip › Figure S1.tif]

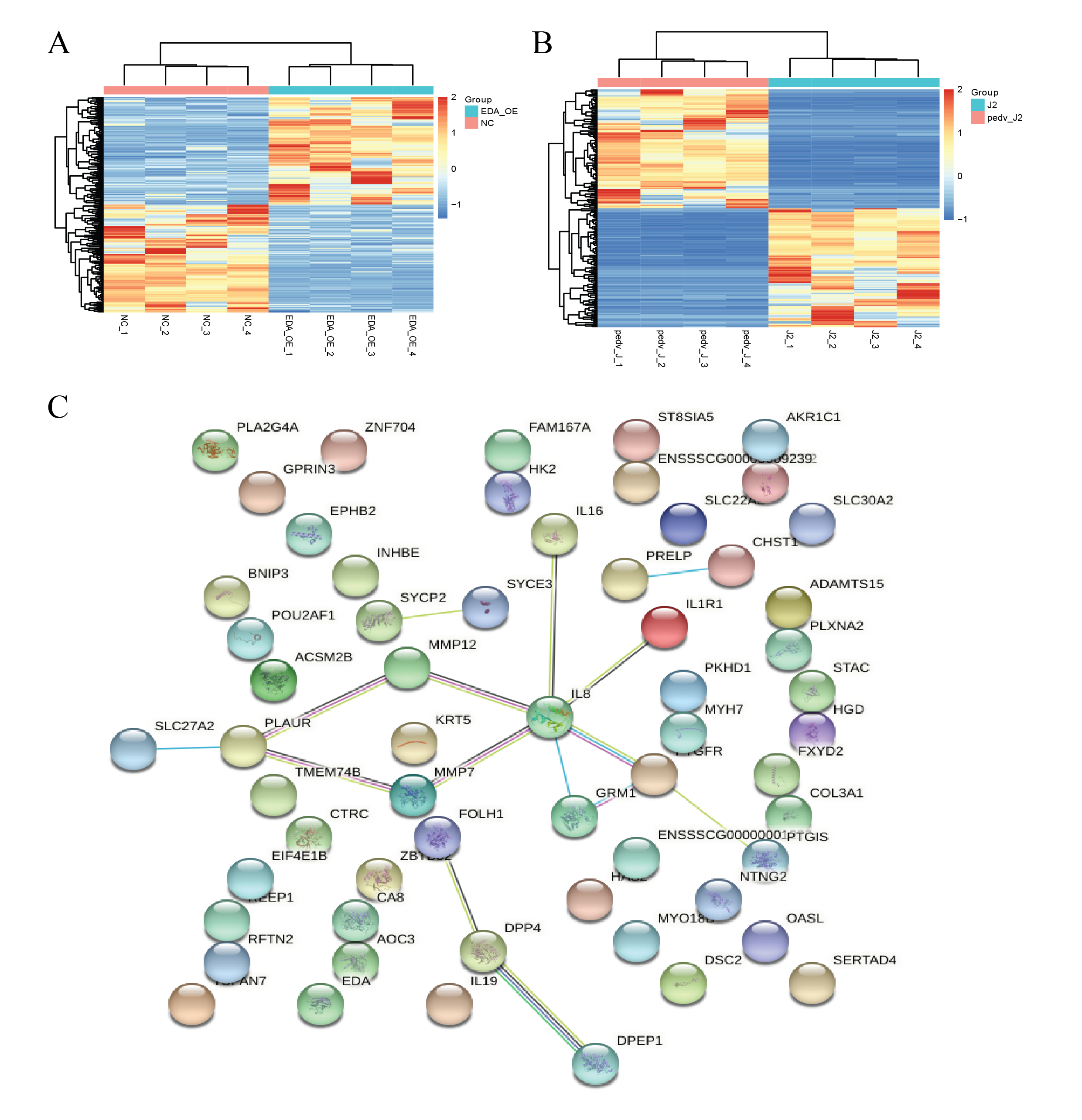

Supplement: Supplementary file 1 [file ijms-22-08133-s001.zip › Figure S2.tif]
